# Conservative management of pelvic fractures in dogs and cats in Algiers: Incidence and long-term clinical outcomes

**DOI:** 10.14202/vetworld.2020.2416-2421

**Published:** 2020-11-12

**Authors:** Ryhan Bouabdallah, Fatima-Zohra Meghiref, Naouelle Azzag, Chabha Benmohand, Wahiba Zenad, Myriem Rebouh

**Affiliations:** 1 Department of Surgery, Higher National Veterinary School, Algiers, Algeria; 2 Research Laboratory Management of Local Animal Resources (GRAL), Higher National Veterinary School, Algiers, Algeria; 3 Department of Clinic, Higher National Veterinary School, Algiers, Algeria

**Keywords:** Cats, dogs, nonsurgical management, pelvic fractures

## Abstract

**Aim:**

We performed a retrospective study to evaluate clinical complications and outcomes associated with non-operative management of pelvic fractures in dogs and cats and described owner satisfaction.

**Materials and Methods:**

Based on radiographic findings and fracture location, case records were classified into two groups. Group 1 included animals with acetabulum involvement that underwent conservative treatment plus femoral head-and-neck excision. Group 2 included animals without acetabulum involvement that underwent conservative treatment only. Compliance with rest instructions, time to locomotion recovery, and the evaluation of persistent lameness were data collected from the questionnaire. The level of satisfaction was classified as excellent, good, or bad. Clinical outcome was evaluated at least 10 months after the fracture.

**Results:**

Pelvic injuries included sacroiliac luxations (59.52%) and ilial body (35.7%), acetabular (21.4%), pubic (21.4%), and ischial (14.28%) fractures alone or combined. According to the owners, the proposed strategy yielded good to excellent outcomes in dogs and cats in this study, with 95.23% of animals regaining full function of their hind limbs. Two dogs had slight chronic lameness, and some degree of gait abnormality persisted.

**Conclusion:**

Because of financial constraints, the chronicity of fractures, or lack of surgical techniques, the surgical treatment of pelvic fractures may not be possible. Non-operative treatment can then be considered to allow the animal to return to acceptable function.

## Introduction

The previous studies have reported that pelvic fractures represent 16-32% of all fractures in dogs and cats [1-5], with this rate being 16% in dogs and 25% in cats [6,7]. Such fractures are associated most commonly with traffic accidents or a fall from a height [1,3,6,8-12].

Pelvic fractures can be treated conservatively or surgically [2,8,13-16]. Surgical intervention should be considered for fractures located in the weight-bearing segment of the pelvis; sacroiliac luxation with displacement compromising the pelvic canal or coxofemoral joint alignment; displacement of acetabular articular surfaces; ilium, ischium, or pubis fractures that cause hip instability; and injuries that cause unilateral or bilateral instability [2,8,13]. Conversely, for animals with minimal hemipelvis displacement and no significant pelvic canal narrowing, stable ilium fractures, and minimally displaced fracture separation of the sacroiliac joint, non-operative treatments are indicated, such as imposed cage rest or moderation of activity for 4-8 weeks [3,8,9,11-18]. In addition, some studies have shown that cats can be treated conservatively if they are ambulatory [13,15]. However, to the best of our knowledge, no study in Algeria has evaluated the conservative management of pelvic fractures in dogs and cats.

We performed a retrospective study to evaluate clinical complications and outcomes associated with non-operative management of pelvic fractures in dogs and cats and described owner satisfaction.

## Materials and Methods

### Ethical approval

The therapeutic procedures were applied in the frame of the surgical consultations of dogs and cats presented to our institution. The animals were kept in good condition by their owners. Therefore, ethical approval was not required.

### Animals

Medical records of dogs and cats admitted to Algiers Higher National Veterinary School (Algeria) between 1998 and 2018 with pelvic fractures that were treated non-operatively were reviewed. Only animals with a follow-up of >10 months postoperatively were included in the study. Incomplete records were not examined.

### Medical records review

Collected data over 20 years included animal signalment, clinical signs, neurologic and radiographic results, initiated treatment, and outcome. At admission, animals were placed under general anesthesia or sedation to obtain lateral and ventrodorsal radiographs of the pelvis. Treatment procedures were decided based on radiographic findings and fracture location. The animals were classified into two groups after analyzing the records. Group 1 (8 animals) included animals with acetabular involvement that underwent conservative treatment with femoral head-and-neck excision. Group 2 (34 animals) included animals without acetabular involvement that underwent conservative treatment only. Experienced academic surgeons performed the surgery.

Post-operative pain management included the intravenous injection of buprenorphine and a prescription for an oral nonsteroidal anti-inflammatory drugs (NSAIDs) medication (meloxicam, Metacam^®^; Boehringer Ingelheim, Germany) for 5 days. Post-operative treatment comprised antibiotic therapy (amoxicillin/clavulanate, Augmentin^®^; GlaxoSmithKline, UK) in Group 1, whereas Group 2 animals received an NSAID (meloxicam, Metacam^®^; Boehringer Ingelheim, Ingelheim am Rhein, Germany) at a dose of 0.1-0.2 mg/kg orally for 15 days and a paraffin laxative to produce soft stools.

### Follow-up

Post-operative clinical follow-up examinations were scheduled at the time of suture removal. Long-term functional status was evaluated through phone call or a written questionnaire completed by the owners. The owner was asked to evaluate using a descriptive scale the treatment results and their satisfaction. The collected data included compliance with rest instructions, time to locomotion recovery, and the evaluation of persistent lameness. The level of satisfaction was classified as excellent (rapid resumption of locomotion without lameness), good (adequate locomotion recovery with slight lameness), or bad (persistent lameness associated with pain), or finally the owner’s dissatisfaction with the results.

### Statistical analysis

In this study, a standardized statistics analysis was applied. Data generated from the clinical records were analyzed using Microsoft Office Excel 2013 (Microsoft Corp., Redmond, WA, USA) and summarized using descriptive statistics. All numerical data were expressed as median and range for continuous variables and as percentages for categorical data. Different parameters, such as age, weight, sex, cause of pelvic fractures, locations, and the different combinations of pelvic fracture and treatment implemented, were studied.

## Results

### Signalment and medical history

We studied 17 dogs and 25 cats, Group 1 included 8 animals (2 dogs and 6 cats) and Group 2 included 34 animals (15 dogs and 19 cats). Affected dog breeds included German Shepherd (n=13), Belgian Malinois (n=1), Braque (n=1), Poodle (n=1), and Rottweiler (n=1). Cat breeds included European shorthair (n=19), Siamese (n=4), Angora (n=1), and Chartreux (n=1). There were 26 intact males and 16 intact females. Median age was 1 year (range, 2 months-9 years), with 32 animals (76%; 71% of dogs and 48% of cats) being <3 years old. Median weight was 21 kg (range, 3-32 kg) for dogs and 3.8 kg (range, 1-6.7 kg) for cats.

Fractures were caused by a traffic accident in 28 cases (66.66%; 8 dogs, 20 cats) or a fall from a height in 14 (33.33%; nine dogs, five cats). The median duration of lameness before presentation was 2 days (range, 12 h-10 days). At clinical examination, no other injuries were noted, but 20% (8/42) of animals exhibited moderate neurologic disorders, and four cats and one dog suffered from constipation. Three cats in Group 2 were ambulatory.

### Radiographic findings

Radiographic finding revealed the following injuries: Oblique ilial body fracture (n=8); combined ipsilateral ilial body (comminuted in one case) and unilateral acetabular (n=2) fractures; unilateral acetabular fracture (n=4); unilateral acetabular fracture with ilial fracture and sacroiliac luxation (n=1); acetabular fracture with ipsilateral ilial fracture, sacroiliac luxation, and ischial fracture (n=1); unilateral acetabular fracture with unilateral sacroiliac luxation (n=1); ilial fracture with sacroiliac luxation (n=1); ilial fracture with sacroiliac luxation and pubic fracture (n=1); ilial fracture with ipsilateral ischial fracture (n=1); unilateral sacroiliac luxation alone (n=11); minimally displaced unilateral sacroiliac luxation with ischial fracture (n=1); unilateral sacroiliac luxation with pubic fracture (n=7); bilateral sacroiliac luxation with pubic and ischial fractures (n=1); and ischial fracture (n=2).

There were 16 cases of pelvic canal narrowing, including 11 cats (seven in Group 2 and four in Group 1) and five dogs (four in Group 2 and one in Group 1). Pelvic canal narrowing was severe (−30%-45%) [19] in eight cats and moderate (−10%-−30%) [19] in three cats, and two dogs from the 4 dogs in Group 2 had moderate narrowing (<30%) and the dog from group 1 had severe pelvic canal narrowing (40%).

### Treatments

All animals underwent conservative management. The 34 animals (80.95%) in Group 2, which included 88% of dogs (15/17) and 80% of cats (21/27), were prescribed 4-6 weeks of activity restriction for dogs and cage therapy for cats. Of the 8 animals (19.04%) in Group 1, the same conservative treatment was applied in combination with the femoral head-and-neck excision. Group 1 included one dog with acetabular fracture, one dog with acetabular fracture and comminuted ilial body fracture, and six cats (two with acetabular fracture, one with acetabular fracture and ilial body fracture, one with acetabular fracture and sacroiliac luxation, one with acetabular fracture and ilial body fracture and ischial fracture, and one with acetabular fracture and ilial body fracture and sacroiliac luxation). The median weight of the dogs and cats that underwent conservative treatment combined with femoral head-and-neck excision was 23.5 and 3.7 kg, respectively.

### Post-operative follow-up

The average time to resuming locomotion was 3 weeks (range, 2-6 weeks) in Group 1 and 3 weeks (range, 1 week-1 month) in Group 2. All animals in Group 1, according to their owners, regained full function of their hind limbs with no complications. For example, an owner reported that his dog (Braque; combined comminuted ilial body and acetabular fractures) resumed its hunting activities approximately 8 months after the traffic accident. Of the 34 animals in Group 2, 32 (94.11%) regained full function of their hind limbs. Two dogs had slight chronic lameness, and some degree of gait abnormality persisted, whereas one cat was less active and had slight difficulties in jumping. Based on the more or less rapid resumption of locomotion and absence of lameness after treatment except for two dogs, the owners were highly satisfied with the clinical evolution of their animals.

## Discussion

A road traffic accident is the most common reported cause of pelvic fractures [4,6,7,20], likely due to the increase in motor vehicle traffic over time [7]. Draffan *et al*. [21] and Vassalo *et al*. [20] reported that this etiology was the most common in dogs. Our study showed that the prevalence of road traffic accidents was higher in cats than in dogs (79% vs. 47%), whereas falling from a great height was a more common cause of injury in dogs than in cats (53% vs. 21%). These differences may be due to that most dogs in our study were kept more commonly on home terraces without protective barriers and that cats were outside of the house more often and thus were more commonly vulnerable to traffic accidents.

Our patient population was predominantly male, including 12 male dogs (71%) and 14 male cats (56%). This finding has been shown in other studies of pelvic injuries among dogs and cats, of which 44-70% have been males [4,22]. German Shepherd dogs and European shorthair cats were the most affected breeds.

A total of 32 animals (76%) were <3 years old, including 12 dogs (28.5%) and 20 cats (48%). In addition, 52% (13/25) of cats were ≤12 months old. Our results agreed with those of previous studies [1,6,8].

In recent studies byHoffberg *et al*. [10] and Gant *et al.*, [23] 57.8% and 67.9% of dogs and cats, respectively, had sacroiliac dislocation. In our study (Table-1), records showed a predominance of sacroiliac luxation (59.52%) in 52.9% and 64% of dogs and cats, respectively, which sometimes was associated with ≥1 pelvic fractures in 30.95% (13/42). Among the sacroiliac luxation cases, 100% in dogs and 96% in cats were unilateral. These results are superior to those obtained in previous findings, showing unilateral sacroiliac luxation in 33.72-77% of cases; nevertheless, they are below the results cited for bilateral sacroiliac luxation (23-75% of cases) [1,4,18,22,24]. Pelvic fractures are associated with sacroiliac luxation in 21-93% of cases in dogs and cats [1,7,9,11]. Conversely, ilial fractures were reported to occur in 20-51% [1,5,7,8,20] and acetabular fractures in 17.5-30.1% of all pelvic fractures [5,7,11]. In our study, ilial body fractures constituted 35.7% of all fractures, whereas acetabular fractures and pubic fracture displayed similar findings (21.42%) followed by ischial fractures (14.28%).

**Table-1:** Number (n) and incidence (%) of each fracture type of 17 dogs and 25 cats.

Fracture site	Dogs, n (%)	Cats, n (%)
Sacroiliac luxation	9 (52.9)	16 (64)
Acetabular fracture	3 (17.6)	6 (24)
Ilium fracture	5 (29.4)	10 (40)
Pubic fracture	5 (29.4)	4 (16)
Ischium fracture	2 (11.76)	4 (16)

In other studies in which computed tomography (CT) scanning was performed, acetabular fractures occurred in 28-30.09% of dogs and 4-17.94% of cats, whereas ilial fractures occurred in 29.09-64% and 12-30.76%, respectively [21,22].

The distribution and number of multiple pelvic fractures were higher in cats than in dogs (Table-2). Seventeen animals (40.5%) exhibited ≥2 pelvic fractures or sacroiliac luxation and ≥1 pelvic fractures. This rate was far below the results obtained in other studies showing that 57.8-95% of animals had multiple pelvic fractures or sacroiliac luxation and pelvic fracture [4,7,10,14,18,20,24,25]. Therefore, a careful evaluation of radiographs should be performed if only one fracture is identified initially [7].

**Table-2:** Number (n) and incidence (%) of fracture sites of 17 dogs and 25 cats.

Lesion	Dogs, n (%)	Cats, n (%)
1	11 (64.7)	14 (56)
2	5 (29.4)	8 (32)
3	1 (5.88)	1 (4)
4	0 (0)	2 (8)
Total	17 (100)	25 (100)

Considering that our study includes 10 cases from as early as 1998, it should be noted that most X-rays were not taken with a digital X-ray machine. Additional oblique views avoiding overlapping images allowed for better diagnosis of acetabular fractures [2,12]. We believe that this is one reason that some fractures may have escaped notice. High-quality radiography is recommended for all pelvic fracture cases and allows a good evaluation of images. In addition, CT is useful to evaluate complex fractures since CT is more precise than a conventional X-ray and because three-dimensional images can provide detailed anatomic information [21,26], particularly for acetabular fractures [21,27].

In some cases, the choice between non-operative and operative treatments can be influenced by economics criteria [28] or the chronicity of fractures or because the patient has regained ambulatory function [13,15]. In our study, conservative treatment is a financially driven choice made by the owners rather than a medical choice, and this was applied for all animals, regardless of fracture location. This choice also was made by the surgeons because three cats (Group 2) with unilateral sacroiliac luxation (n=2) and unilateral sacroiliac luxation with ischial fracture (n=1) were ambulatory. This treatment has been applied for 16-100% of dogs [9,18,23] and 11-100% of cats [7,8,16-18]. According to some investigators [2,7,15,29], conservative treatment also has been used in association with femoral head-and-neck excision (Group 1) in 12% of fractures in dogs and 24% in cats among cases involving the acetabulum. A notable exception was in a 2-month-old puppy (Group 2); this skeletally immature animal had an acetabular fracture that showed no ventrodorsal displacement and was treated with marked restriction of activity for 3-4 weeks [2].

The pelvis is surrounded by large muscles that are very effective in stabilizing fractures and that provide a rich extraosseous blood supply. This explains the rapid formation of the stabilizing callus and high rate of the bone union following conservative treatment of certain pelvic fractures [2,13,15]. Several investigators have reported that acetabular fracture stabilization should not be based solely on fracture location and should be managed surgically [8,12,20,30,31]. However, excision arthroplasty can be considered a salvage operation by removing bony contact between the acetabulum and femoral head. Indeed, this can be a means of improving the quality of life for many dogs and cats by helping to reduce pain and avoid complications of chronic osteoarthrosis of the hip joint [2,15,29,32,33].

It also should be emphasized that in our study, the instructions regarding cage restraint or activity restriction and physiotherapy, which can be very useful after conservative management, were not strictly followed by owners once the animals started to walk. Regardless of the type of fracture, cats recovered better than dogs, according to their owners. Cats (young or low weight animals) recovered faster than larger dogs after conservative treatment of pelvic fractures or after femoral head-and-neck excision [7,32]. Indeed, post-operative results after femoral head-and-neck excision in dogs weighing <18-20 kg were satisfactory [32]. In our study, the median weight of the dogs that underwent conservative treatment and femoral head-and-neck excision was 23.5 kg. Moreover, some investigators reported a good to excellent outcome with femoral head-and-neck excision and a generally favorable prognosis for conservative management of pelvic fractures [2,6-8,29,32-34]. Reportedly, 64-75% of dogs and 82.35% of cats presenting with pelvic fractures had good to excellent outcomes with conservative treatment [8,14] when the candidates for this treatment were chosen [16,17]. Although pelvic fractures were historically managed conservatively in cats [3,11]. Long-term complications appear and affect the quality of life of the animals [11] and can cause persistent abnormal gait in dogs [20]. This information was reported on two dogs in our study.

Pelvic narrowing >45-50% (respectively, in cats and dogs) potentially could still be a risk for malunion or pelvic canal narrowing that can result in constant or intermittent constipation after conservative treatment [7,12,19,35,36]. Intermittent constipation has been reported in two cats in Group 2. No other patient suffered constipation. Because of the risk of dystocia in females with severe pelvic narrowing (four cats), an ovariohysterectomy has been requested by the cats’ owners. Because the latter animals had free access to the outside, the owners did not wish a possible gestation. Neither of the two female dogs with pelvic narrowing (<50%) experienced gestation.

On initial clinical examination, 20% (8/42) of the animals in our study exhibited neurologic deficit, which subsequently resolved within 10 days-2 weeks. Neurologic deficits were recorded in 6-28% of dogs and cats with pelvic fracture [1,4,7,11].

There are limitations to this study. First, the number of cases was small, which can be explained by the following reasons. Our study was limited to the region of Algiers. Annually, we received in the department of surgery an average of 120 cases of fractures of all types and pelvic fractures accounted for barely 20% of these fractures. Similar studies also were conducted using a similarly small sample size [11,16,17]. The old records have not been computerized, and our school has changed site, so a number of these paper files have been lost. Moreover, some files are incomplete and could not be exploited for the study. Finally, it should be noted that many owners did not present their animals for consultation after trauma as soon as the animals started walking. Because of the retrospective nature, patients could not be examined and we were unable to obtain long-term radiographs for all patients. Furthermore, the complication of megacolon related to constipation reported by two owners’ cats could not be evaluated. This study allowed a qualitative evaluation of the locomotor function based on patient quality of life perceived by its owner.

Most veterinary orthopedic surgeons would consider most pelvic fractures in dogs and cats to be candidates for surgery. However, in some cases, this treatment cannot be performed because of financial constraints or the chronicity of fractures or because veterinarians are not orthopedic surgeons. Some animals in this study were referred by their veterinarians who were reluctant to institute conservative treatment. We would like to encourage veterinary practitioners in Algeria during the decision-making process to choose this treatment option for pelvic fractures when surgery cannot be considered.

## Conclusion

In general, our results indicated excellent outcomes in cats and satisfactory outcomes in dogs, regardless of the number and location of the fractures. Conservative treatment remains a good option for managing pelvic fractures; because of the satisfactory results provided in our study, this treatment can be considered to allow the animal to return to normal function.

## Authors’ Contributions

RB designed the study, performed the surgical consultations, radiographic analysis and surgery, as well as to analysis and interpretation of data and drafted the manuscript. FM acquired, analyzed the data, and performed statistical analysis. NA revised and commented on the manuscript from the critical point of view. CB and WZ surgical consultations and radiographic analysis. MR surgical consultations, radiographic analysis, surgery, and revised the manuscript. All authors read and approved the final manuscript.
